# Antimicrobial resistance in *Mannheimia haemolytica* from diseased cattle: phenotypic and genotypic insights over five years (2018–2022)

**DOI:** 10.1186/s12864-026-12783-y

**Published:** 2026-07-25

**Authors:** Selma Burciaga, Laura Alt, Kathe Bjork, Beth Harris, Christina Loiacono, Jessica Hicks

**Affiliations:** 1 Oak Ridge Institute for Science and Education (ORISE), Animal and Plant Health Inspection Service (APHIS) Research Participation Program, Oak Ridge, TN USA; 2 United States Department of Agriculture, Animal and Plant Health Inspection Service (APHIS), Veterinary Services, Diagnostics & Biologics, National Veterinary Services Laboratories (NVSL), National Animal Health Laboratory Network (NAHLN), Ames, IA USA; 3 United States Department of Agriculture, Animal and Plant Health Inspection Service (APHIS), Veterinary Services, Diagnostics & Biologics, National Veterinary Services Laboratories (NVSL), Ames, IA USA; 4 United States Department of Agriculture, National Institute of Food and Agriculture (NIFA), Institute of Food Production and Sustainability, Kansas City, MO USA

**Keywords:** *Mannheimia haemolytica*, Antimicrobial resistance, MDR, Genomics, Genotypes, Phenotypes, Subtypes, Bovine respiratory disease

## Abstract

**Background:**

*Mannheimia haemolytica* is a gram-negative bacterium that typically resides in the upper respiratory tract of healthy cattle but can invade the lower respiratory tract in immunocompromised cattle, contributing to bovine respiratory disease (BRD). The rise of antimicrobial resistance (AMR) in *M. haemolytica* strains, especially those linked to BRD, is a growing concern. This study aimed to investigate AMR phenotypes and genotypes in *M. haemolytica* isolates collected during the NAHLN AMR Pilot Project (2018–2022) and assess the use of a previously developed single nucleotide polymorphism genotyping scheme to classify strains and identify associations between genotypes, AMR genes, quinolone point mutations (QPMs) and mobile genetic elements in diseased cattle.

**Results:**

Of 2743 isolates tested for antimicrobial susceptibility, 66.7% (1830/2743) were pan-susceptible, while 33.3% (913/2743) showed resistance to at least one antimicrobial, primarily tetracyclines. Among the 848 sequenced isolates, 36.7% (311/848) harbored AMR genes and/or QPMs. Of these, 59.8% (186/311) were phenotypically classified as multidrug-resistant; commonly detected AMR genes included *aadA*, *ant(2’’)-Ia*, *bla*_OXA−2_, *mph*(E), *msr*(E), and *tet*(H). The study found that 88.3% (749/848) of isolates belonged to genotype 2, a group previously associated with BRD and AMR. Notably, 95.8% (298/311) of isolates harboring AMR genes were from genotype 2. Although most clinical isolates of *M. haemolytica* were pan-susceptible and lacked AMR genes, a substantial proportion of isolates exhibited AMR, with a high degree of concordance between genotype and phenotype across all antimicrobials tested. Most clinical isolates (749/848, 88.3%), primarily obtained from the cattle lungs rather than the upper respiratory tract, were classified as genotype 2. These isolates harbored a T4SS integrative and conjugative element (ICE, 283/848, 33.4%) as well as an integrative and mobilizable element (IME, 59/848, 7.0%) containing AMR genes.

**Conclusions:**

This study highlights a strong association between genotype 2 *M. haemolytica* and AMR, with a significant prevalence of multidrug-resistant strains. The findings underscore the importance of genotypic characterization and whole-genome sequencing (WGS) for monitoring AMR in *M. haemolytica* and informing future management strategies for BRD.

**Supplementary Information:**

The online version contains supplementary material available at https://doi.org/10.1186/s12864-026-12783-y.

## Background

Bovine respiratory disease (BRD) is a significant global animal health concern and a major economic burden to cattle industries. In the United States alone, BRD costs the cattle industry approximately one billion dollars annually from death loss, treatment costs, and reduced feed efficiency and carcass quality [1–3]. The etiology of BRD is multifactorial, resulting from complex interactions between pathogens, host factors, and the environment [4]. Various viral and bacterial pathogens are implicated in BRD, including bovine herpesvirus 1 (BHV1), parainfluenza virus type 3 (PI3), bovine respiratory syncytial virus (BRSV), bovine viral diarrhea virus (BVDV), *Mannheimia haemolytica* (*M. haemolytica*), *Pasteurella multocida* (*P. multocida*), *Mycoplasma bovis* (*M. bovis*), and *Histophilus somni* (*H. somni*). Among these, *M. haemolytica* is a primary contributor to BRD in feedlot cattle and enzootic pneumonia in both beef and dairy cattle [2, 5].

*M*. *haemolytica* is the most frequently isolated pathogen from the lungs of cattle with respiratory disease, where the primary cause of death is severe, acute, hemorrhagic fibrinous pleuropneumonia [4, 6, 7]. *M*. *haemolytica* is a gram-negative, opportunistic pathogen that typically inhabits the upper respiratory tract of healthy cattle. However, it can invade the lower respiratory tract of immunocompromised cattle following viral infections, stress, or other risk factors [3, 8]. Infected cattle commonly show symptoms such as fever, nasal discharge, coughing, and weight loss due to depression [9].

In feedlots, BRD is managed using a combination of strategies, including segregation of high- and low-risk cattle, the administration of vaccines targeting both viral and bacterial respiratory pathogens, and the use of antimicrobials for metaphylaxis or direct treatment of infected cattle [6, 10]. Commonly used antimicrobials for the treatment of *M. haemolytica* and other BRD-associated pathogens in the United States include macrolides (e.g., gamithromycin, tulathromycin, and tilmicosin), fluoroquinolones (e.g., danofloxacin and enrofloxacin), beta-lactams (e.g., ceftiofur), phenicol (e.g., florfenicol) and tetracyclines (e.g., chlortetracycline and oxytetracycline) [11–16]. However, *M. haemolytica* exhibits a wide range of susceptibility to these drugs, with many strains isolated from cattle showing reduced susceptibility over the past few decades [17, 18]. Strains isolated from the lower respiratory tracts of cattle with clinical disease often exhibit elevated antimicrobial resistance (AMR) and multidrug-resistance (MDR), particularly in cattle subjected to multiple antibiotic treatments, compared to strains from healthy cattle [19–24]. This suggests that antimicrobial use in cattle may contribute to the emergence of resistant *M. haemolytica* strains, raising concerns about the continued effectiveness of these treatments.

The emergence of AMR in *M. haemolytica* is strongly associated with integrative and conjugative elements (ICEs), which are mobile genetic elements (MGEs) that facilitate the horizontal transfer of AMR genes [25, 26]. These ICEs, including ICE*Mh1* in *M. haemolytica* and ICE*Pmu1* in *P. multocida*, play a crucial role in the horizontal spread of AMR, conferring resistance to the antimicrobials commonly used in BRD treatment [26–28]. ICE*Mh1* contains six AMR genes, including those for aminoglycosides/aminocyclitol (*aadA25*, *ant(2”)-Ia*), beta-lactams (*bla*_OXA−2_), macrolides (*msr*(E), *mph*(E)), and tetracycline (*tet*(H)) resistance [27]. ICE*Pmu1* harbors the same genes but also contains AMR genes for sulfonamides (*sul2*), phenicol (*floR*), and additional aminoglycoside and macrolide resistance genes (*aph(3’)-Ia, aph(3”)-Ib, aph(6)-Id, erm(42)*) [28]. Homologs of ICE*Pmu1* and ICE*Mh1* have been identified in *M. haemolytica* strains, with variations in the quantity and diversity of AMR genes [8].

Strain typing using capsular phenotypes and pulsed-field gel electrophoresis (PFGE) has revealed that *M. haemolytica* consists of distinct subspecies or strain types, each with varying associations to BRD. For example, strains with A2 or A4 capsular serotypes are more commonly found in the upper respiratory tracts of healthy cattle, while A1 or A6 serotypes are more often isolated from the lungs and nasopharynx of diseased cattle [2, 29]. Additionally, Clawson et al. developed a single nucleotide polymorphism (SNP) genotyping scheme that identified two major genotypes of *M. haemolytica*, each with several subtypes [8]. Genotype 1 is predominantly composed of non-clinical isolates from the nasopharynx of healthy cattle without respiratory disease and has been identified in three U.S. states. In contrast, genotype 2, has been detected across multiple U.S. states and Canada, with isolates predominantly recovered from the respiratory tract of diseased cattle [8, 30]. ICEs carrying AMR genes are primarily associated with genotype 2 strains. Importantly, several studies have established that *M. haemolytica* isolates harboring these MGEs show strong associations with respiratory disease, highlighting the clinical significance of ICE-mediated resistance [8, 26, 31, 32].

The aim of our study was to investigate the phenotypic and genotypic AMR profiles of *M. haemolytica*, assess the concordance between phenotypic susceptibility and resistance genotypes, identify associated MGEs, and use the SNP typing method to explore genetic relationships in *M. haemolytica* samples from clinical veterinary diagnostic submissions collected between 2018 and 2022 across the United States as part of the National Animal Health Laboratory Network (NAHLN) AMR Pilot Project.

## Methods

### Laboratory enrollment

Laboratories participating in the NAHLN AMR Pilot Project were enlisted on an annual basis via a request to the Animal and Plant Health Inspection Service (APHIS) NAHLN. To maximize representation of isolates surveyed in the United States, factors such as geographic and animal population representativeness were considered. Enrolled laboratories submitted antimicrobial susceptibility testing (AST) and/or whole-genome sequencing (WGS) data from 2018 to 2022. A total of 31 laboratories participated across the following states: Alabama, California, Colorado, Florida, Georgia, Illinois, Indiana, Iowa, Kansas, Kentucky, Louisiana, Michigan, Minnesota, Mississippi, Missouri, Nebraska, New York, North Carolina, North Dakota, Ohio, Pennsylvania, South Dakota, Tennessee, Texas, Washington, Wisconsin, and Wyoming.

### Isolate selection

We received data from a total of 2743 *M. haemolytica* isolates collected from cattle. Isolates were selected by participating laboratories from routine diagnostic case submissions evaluated with AST. Bacterial identification followed current protocols established at participating facilities, using either matrix-assisted laser desorption ionization time-of-flight mass spectrometry (MALDI-TOF MS) instrument [33] or conventional phenotypic techniques [34]. The inclusion criteria for all isolates were as follows: confirmed identification to the genus and species level using standard veterinary microbiology laboratory techniques, association with clinical respiratory infection or relevant diagnostic findings (including postmortem examinations), and selection from unique animal sources (e.g., one isolate per year from the same herd/farm/owner/household). To protect personally identifiable information associated with isolates, each participating laboratory was assigned a unique identifier by NAHLN. The following data and metadata were collected for each isolate: the minimum inhibitory concentration (MIC) values of all antimicrobials tested, bacterial organism (genus/species), date of isolation, animal species, state of origin of animal, source tissue, final confirmed diagnosis, and initial reason for submission (e.g., suspected diagnosis and clinical or pathological observations). Isolates were selected for sequencing at different time points to avoid bias from sampling multiple isolates within the same month.

Animals were not euthanized for the purpose of this study. Any euthanasia performed was solely at the discretion of the animal’s owner and the licensed veterinarian responsible for its care.

### Antimicrobial susceptibility testing

AST was conducted using the Sensititre™ broth microdilution platform (Thermo Fisher Scientific, Waltham, MA), with commercially available Sensititre™ microdilution plates (BOPO6F and BOPO7F, Thermo Fisher Scientific), following the manufacturer’s instructions. Table 1 lists the twelve antimicrobials included across the two plates, each with veterinary breakpoints for *M. haemolytica* in cattle established by the Clinical Laboratory Standards Institute (CLSI) Vet01S 7th edition (2024) [35]: ceftiofur (XNL), danofloxacin (DAN), enrofloxacin (ENR), florfenicol (FFN), gamithromycin (GAM), tildipirosin (TIP), tilmicosin (TIL), tulathromycin (TUL), ampicillin (AMP), penicillin (PEN), spectinomycin (SPE), oxytetracycline (OXY) and tetracycline (TET). These antibiotics represent 6 different antibiotic classes: beta-lactams (XNL, AMP, PEN), fluoroquinolones (DAN, ENR), phenicol (FFN), macrolides (GAM, TIP, TIL, TUL), aminocyclitol (SPE), and tetracyclines (TET, OXY). Susceptibility to TET, GAM and TIP was evaluated by only laboratories using the BOPO7F plate. The frequency and percentage of AMR for each antimicrobial with established breakpoints were reported based on the total number of isolates tested. Isolates were classified as MDR if they were resistant to three or more antimicrobial classes.

**Table 1:** Clinical and Laboratory Standards Institute (CLSI) Vet01S 7th edition minimum inhibitory concentration (MIC) breakpoints for M. haemolytica and associated antimicrobial resistance (AMR) genes and quinolone (QPM) in isolates from this study

Antimicrobial Class		Antimicrobial Agent	CLSI Established Breakpoints (µg/mL)			AMR Genes and QPMs
			Susceptible (S)	Intermediate (I)	Resistant (*R*)	
Aminocyclitol		Spectinomycin (SPE)	≤ 32	64	≥ 128	*aadA*, *ant(2”)-Ia*, *aph(3”)-Ib*, *aph(3’)-Ia*, *aph(3’)-IIIa*, *aph(6)-Id*
Aminoglycosides		Gentamicin (GEN)^a^	-	-	-	*aadA*, *ant(2”)-Ia*, *aph(3”)-Ib*, *aph(3’)-Ia*, *aph(3’)-IIIa*, *aph(6)-Id*
		Neomycin (NEO)^a^	-	-	-	
Β-Lactams	Penicillins	Ampicillin (AMP)	≤ 0.03	0.06–0.12	≥ 0.25	*bla*_OXA−1_, *bla*_ROB−1_
		Penicillin (PEN)	≤ 0.25	0.5	≥ 1	
	Cephems	Ceftiofur (XNL)	≤ 2	4	≥ 8	-
Quinolones and Fluoroquinolones		Danofloxacin (DAN)	0.25	0.5^*^	≥ 1	gyrA_D87N, gyrA_D87G, gyrA_S83F, gyrA_S83Y, parC_E89K
		Enrofloxacin (ENR)	0.25	0.5-1^*^	≥ 2	
Folate Pathway Antagonists		Sulphadimethoxine (SDM)^a^	-	-	-	*dfrA14*, *sul2*
		Trimethoprim/Sulfamethoxazole (SXT)^a^	-	-	-	
Lincosamide		Clindamycin (CLI)^a^	-	-	-	-
Macrolides		Gamithromycin (GAM)	≤ 4	8	≥ 16	*erm*(42), *mph*(E), *msr*(E)
		Tildipirosin (TIP)	≤ 4	8	≥ 16	
		Tilmicosin (TIL)	≤ 4	8	≥ 32	
		Tulathromycin (TUL)	≤ 16	32	≥ 64	
		Tylosin tartrate (TYLT)^a^	-	-	-	
Phenicols		Florfenicol (FFN)	≤ 2	4	≥ 8	*floR*
Pleuromutilin		Tiamulin (TIA)^a^	-	-	-	-
Tetracyclines		Chlortetracycline (CTET)^a^	-	-	-	*tet*(H), *tet*(T)
		Oxytetracycline (OXY)^b^	≤ 2	4	≥ 8	
		Tetracycline (TET)	≤ 2	4	≥ 8	

^*^ Intermediate (I) classification for fluoroquinolones indicates decreased susceptibility

^a^Antimicrobials with no established CLSI breakpoints in cattle

^b^Oxytetracycline antimicrobials breakpoints were extrapolated from tetracycline breakpoints

### DNA extraction and whole-genome sequencing

A subset of 848 isolates was randomly selected for WGS to identify AMR genes and quinolone point mutations (QPMs) associated with phenotypic AMR. Participating laboratories used their own sequencing platforms or submitted isolates to the National Veterinary Services Laboratories (NVSL) for DNA extraction and WGS. Laboratories selected DNA extraction methods for *M. haemolytica*, and the DNA concentration was quantified with a fluorometer. Nineteen labs with sequencing capabilities and the NVSL used Illumina platforms, including MiSeq, iSeq100, NextSeq 1000, and NextSeq 2000 (Illumina, San Diego, CA), for paired-end sequencing. These labs primarily used the Illumina DNA Prep kit followed by the Illumina Nextera XT kit for library preparation, in accordance with the manufacturer’s instructions. Two laboratories used the Ion GeneStudio S5 and Nanopore GridION sequencing platforms for single-end sequencing, along with the IonXpress Plus Fragment and Nanopore Ligation Sequencing Kits, respectively.

### Bacterial identification, genome assembly, and antimicrobial resistance gene detection

The draft genomes were processed using an in-house AMR pipeline, a collection of custom scripts that integrate open-source tools for bacterial identification, *de novo* assembly, and AMR gene detection. *M. haemolytica* was identified using Kraken with a RefSeq complete genomes database v2.1.0 [36] and validated phenotypically and genotypically. Genomes were assembled using SPAdes v3.13.0 [37]. AMR gene detection was carried out on assemblies using AMRFinderPlus v3.11.2 [38] (with default parameters as *M. haemolytica* is not covered by the organism option) and ABRicate v0.9.8 (Seemann T, Abricate, Github https://github.com/tseemann/abricate), referencing the National Center for Biotechnology Information’s (NCBI) curated Reference Gene Database and the ResFinder database [39]. Precedence was given to the AMRFinderPlus identifications, while ABRicate output was used to corroborate those AMRFinderPlus output and to explore partial gene matches that may have occurred due to the fragmentation of the short-read assemblies. AMR genes (Table 1) were identified as present if they met the criteria of ≥ 70% nucleic acid identity and ≥ 95% reference gene coverage. Sequence quality required an average Q score ≥ 30, with at least 80% of reads meeting this threshold. Assembly coverage was calculated as total bases in raw reads divided by assembly length, and all assemblies required ≥ 30x coverage.

### Identification of T4SS integrative and conjugative element and integrative and mobilizable element sequences

To identify homologs of previously characterized type IV secretion system (T4SS) ICEs harboring AMR genes, namely ICE*Mh1* and ICE*Pmu1*, vSNP [40] was used to align *M. haemolytica* FASTQ files to the ICE*Pmu1* FASTA reference (NCBI accession no. CP003022) (Supplemental file 1). Coverage and mean depth of T4SS-ICE-defining and AMR regions were determined using SAMtools v1.12 [41], with coordinates from the ICEberg 3.0 database via ICEfinder (Table 2) [42]. T4SS-ICE-defining regions included genes encoding tyrosine recombinase-2 family protein (*int2*), integrative conjugative element ParB family protein (*parB*), integrative conjugative element relaxase (*rel1*), DNA topoisomerase III (*topB*), and T4SS components such as TraC, TraD, TraG, pilL, TraU, and TrbI [31, 43]. A T4SS-ICE was considered present if, these ICE-defining genes had ≥ 80% coverage and ≥ 30x mean depth; otherwise, it was defined as absent. AMR region 1 (amr1; *aph(3’)-Ia, aph(3”)-Ib, aph(6)-Id, sul2, floR, and erm*(42)) and AMR region 2 (amr2; *aadA25*, *ant(2”)-Ia*, *bla*_OXA−2_, *msr*(E), *mph*(E), and *tet*(H)) were considered present if coverage was ≥ 80%, with lower coverage suggesting gene variation.

**Table 2:** Type 4 Secretion System (T4SS) integrating conjugative element (ICE)-defining regions and antimicrobial resistance (AMR) regions in ICEPmu1 reference

ICE Targets	Size (BP)	Product	ICEfinder Coordinates*
*int1*	900	Tyrosine recombinase-1 family protein	275,603–276,502
*int2*	765	Tyrosine recombinase-2 family protein	292,568–293,332
*parB*	1647	Integrating conjugative element ParB family protein, PFGI-1 class	351,398–353,044
*rel1*	1980	Integrating conjugative element relaxase, PFGI-1 class	293,488–295,467
*topB*	2052	DNA topoisomerase III	326,566–328,617
T4SS components	21,273	TraC, TraD, TraG, pilL, TraU, and TrbI	301,851–323,124
AMR region 1	11,524	*aph(3’)-Ia*, *aph(3”)-Ib*, *aph(6)-Id*, *sul2*, *floR*, and *erm*(42)	279,777–291,301
AMR region 2	8914	*aadA*, *ant(2”)-Ia*, *bla*_OXA−2_, *msr*(E), *mph*(E), and *tet*(H)	336,102–345,016

*ICE*Pmu1* FASTA reference (NCBI accession no. CP003022)

Similarly, vSNP [40] was used to align *M. haemolytica* FASTQ files to a FASTA reference (NCBI accession no. JBNXNN000000000) derived from a draft genome in our dataset that contains a fully assembled integrative and mobilizable element (IME) harboring *bla*_ROB−1_ and *estT* on a single contig (Supplemental file 1). This contig was analyzed with ICEfinder (ICEberg 3.0) to identify the IME and its coordinates. Coordinates were used with SAMtools [42] to assess coverage (≥ 80%) and mean depth (≥ 30x) of the IME-defining and AMR-associated genes (Table 3). The IME was considered present if it contained IME-defining genes encoding MOBP1 relaxase (*rel*) and IS30 family transposase ISApl1 (*int*).

**Table 3:** Integrative and mobilizable element (IME)-defining genes and antimicrobial resistance (AMR) genes in bla_ROB-1_ and estT contig reference

IME Targets	Size (BP)	Product	ICEfinder coordinates*
*rel*	1407	MOBP1	151–1557
*mbeC*	306	Mobilization protein MbeC	1554–1859
*bla*_ROB−1_	918	Beta-lactamase, blaROB-1	3325–4242
*int*	924	IS30 family transposase ISApl1	5539–6462
*estT*	849	α/β-hydrolase	4325–5173

*IME originated from a draft genome in our collection (NCBI accession no. JBNXNN000000000)

### Single nucleotide polymorphism analyses and phylogenetic relationships

vSNP was used to construct SNP-based phylogenetic trees for genotype 1 and genotype 2 using the Randomized Axelerated Maximum Likelihood (RAxML) with GTR-CATI model of evolution and reference genome USDA-ARS-USMARC-183 (NCBI accession no. CP004752.1) [40]. Genotyping SNPs were initially based on those proposed by Clawson et al. [8, 30], and refined by replacing SNPs located within MGEs or indel regions with core-genome SNPs shared across genomes within each genotype. Regions indicative of MGEs, indels, and repeats were excluded to ensure high-quality genomic SNPs and construction of a core-genome tree. Default vSNP quality threshold filters (SNP quality value ≥ 150) were applied. To validate genotyping, a subset of Clawson et al. [19] genotype 1 (*n* = 21) and genotype 2 isolates (*n* = 52) was randomly selected for inclusion in the phylogenetic analysis. The reference genome was evaluated with PHAster to identify phages [44]. The vSNP output was subsequently used to identify SNPs associated with reduced susceptibility to quinolone antibiotics, i.e. QPMs. Each genome was specifically screened for the following mutations: S83F, D87G, D87N, S83Y, and S88R in *gyrA*; and S80I and E89K in *parC* [45–48].

### Statistical analysis

Descriptive statistics (counts and proportions) were calculated for AMR phenotypic and genotypic profiles, such as pan-susceptibility, resistance to each antimicrobial, and MDR. Proportions for metadata such as collection year and geographical origin, were also computed. Temporal trends in AMR, MDR, and pan-susceptibility across the five-year pilot project (2018–2022) were evaluated using the two-sided Cochran-Armitage test for trend, with P-values < 0.05 considered statistically significant. Significant results indicate a linear trend (increasing or decreasing), while non-significant results suggest no meaningful change.

Concordance and Kappa values were used to assess the agreement between phenotypic AMR profiles and the presence of AMR genes and QPMs within each antimicrobial class [49, 50]. Kappa significance was evaluated using the Z-test statistic [51]. This analysis focused on antimicrobials with CLSI-defined breakpoints for cattle and *M. haemolytica*. For QPMs, concordance included isolates intermediate or resistant to fluoroquinolones, as QPMs do not directly confer resistance but instead contribute to reduced susceptibility to fluoroquinolones [52, 53].

## Results

### Summary of isolate collection

A total of 2743 *M. haemolytica* isolates from clinical cases were submitted during the five-year pilot project: 429 (15.6%) isolates in 2018, 613 (22.3%) in 2019, 563 (20.5%) in 2020, 582 (21.2%) in 2021, and 556 (20.3%) in 2022. Most isolates were collected from cattle lungs (2423/2743, 88.3%), followed by nasal passages or nasopharynx (223/2743, 8.13%), and other tissues, organs, or pooled sources (92/2743, 3.5%); five isolates had undetermined sources. Geographically, submissions were predominantly from the Midwest (1765/2743, 64.3%), with fewer from the South (420/2743, 15.3%), West (366/2743, 13.3%), and Northeast (192/2743, 7.0%) regions, as defined by the U.S. Census Bureau (Supplemental file 1).

### Antimicrobial susceptibility phenotypes

**Fig. 1:**
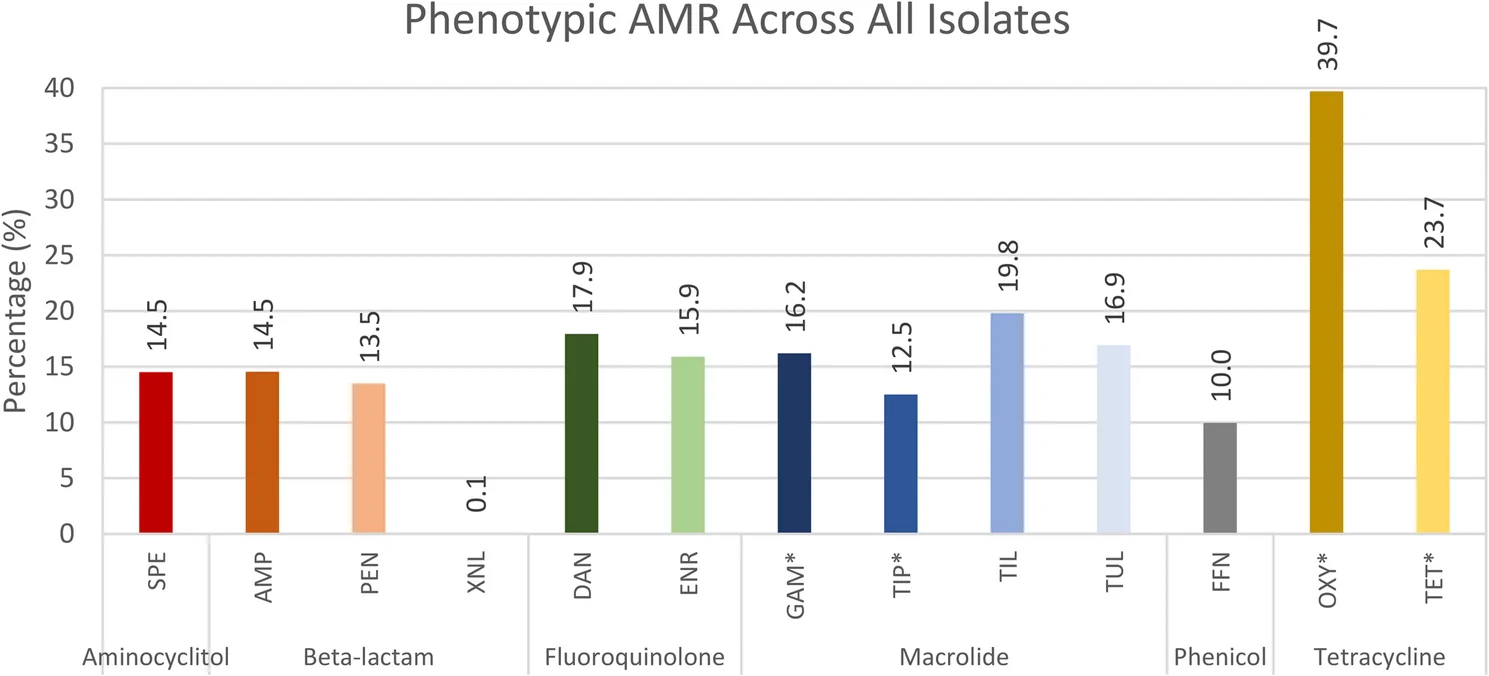
Phenotypic antimicrobial resistance (AMR) across all isolates (*n* = 2743). Bar graph showing the percentage of resistance (y-axis) across various antimicrobials (x-axis), limited to those with CLSI breakpoints. ceftiofur (XNL), danofloxacin (DAN), enrofloxacin (ENR), florfenicol (FFN), gamithromycin (GAM), tildipirosin (TIP), tilmicosin (TIL), tulathromycin (TUL), ampicillin (AMP), penicillin (PEN), spectinomycin (SPT), and tetracycline (TET). *Percentages based on total tested; GAM, TET, and TIP were tested on BOPO7F (*n* = 2008) and oxytetracycline (OXY) tested on BOPO6F (*n* = 735)

AST was performed using Sensititre™ plates BOPO7F (2008/2743, 73.2%) or BOPO6F (735/2743, 26.8%). Most *M. haemolytica* isolates were pan-susceptible (1830/2743, 66.7%), while one-third (913/2743, 33.3%) exhibited resistance to at least one antimicrobial (Fig. 1). Among these, the majority (663/913, 72.6%) were resistant to more than one antimicrobial (Supplemental file 1).

The predominant resistance phenotypes were observed in the tetracycline class (768/2743, 28.0%), with resistance to TET (476/2008, 23.7%) or OXY (292/735, 39.7%) (Fig. 1). Macrolide resistance was also widespread, affecting one fifth of isolates (560/2743, 20.4%). Among isolates tested for all four macrolides on the BOPO7F plate (*n* = 2008), 10.9% (219/2008) showed resistance to all four drugs (GAM, TIP, TIL, and TUL). Resistance to TIL was most common overall (543/2743, 19.8%), followed by TUL (464/2743, 16.9%), likely because they were also tested on the BOPO6F (*n* = 735), whereas GAM (325/2008, 16.2%) and TIP (250/2008, 12.5%) were not, and therefore appeared slightly less prevalent (Fig. 1).

Resistance to other antimicrobial classes ranged from 10% to 18% (Fig. 1). Fluoroquinolone resistance was also notable (493/2743, 17.9%), with most isolates resistant to both DAN and ENR (435/2743, 15.9%), while resistance to a single fluoroquinolone was rare (DAN 57/2743, 2.1%; ENR 1/2743, 0.04%). Similar proportions of resistance were observed for SPE and AMP (398/2743, 14.5%), with most AMP-resistant isolates also resistant to PEN (336/2743, 12.3%). Less common resistance was seen for PEN (370/2743, 13.5%), FFN (273/2743, 10%), and XNL (3/2743, 0.11%) (Fig. 1).

**Fig. 2:**
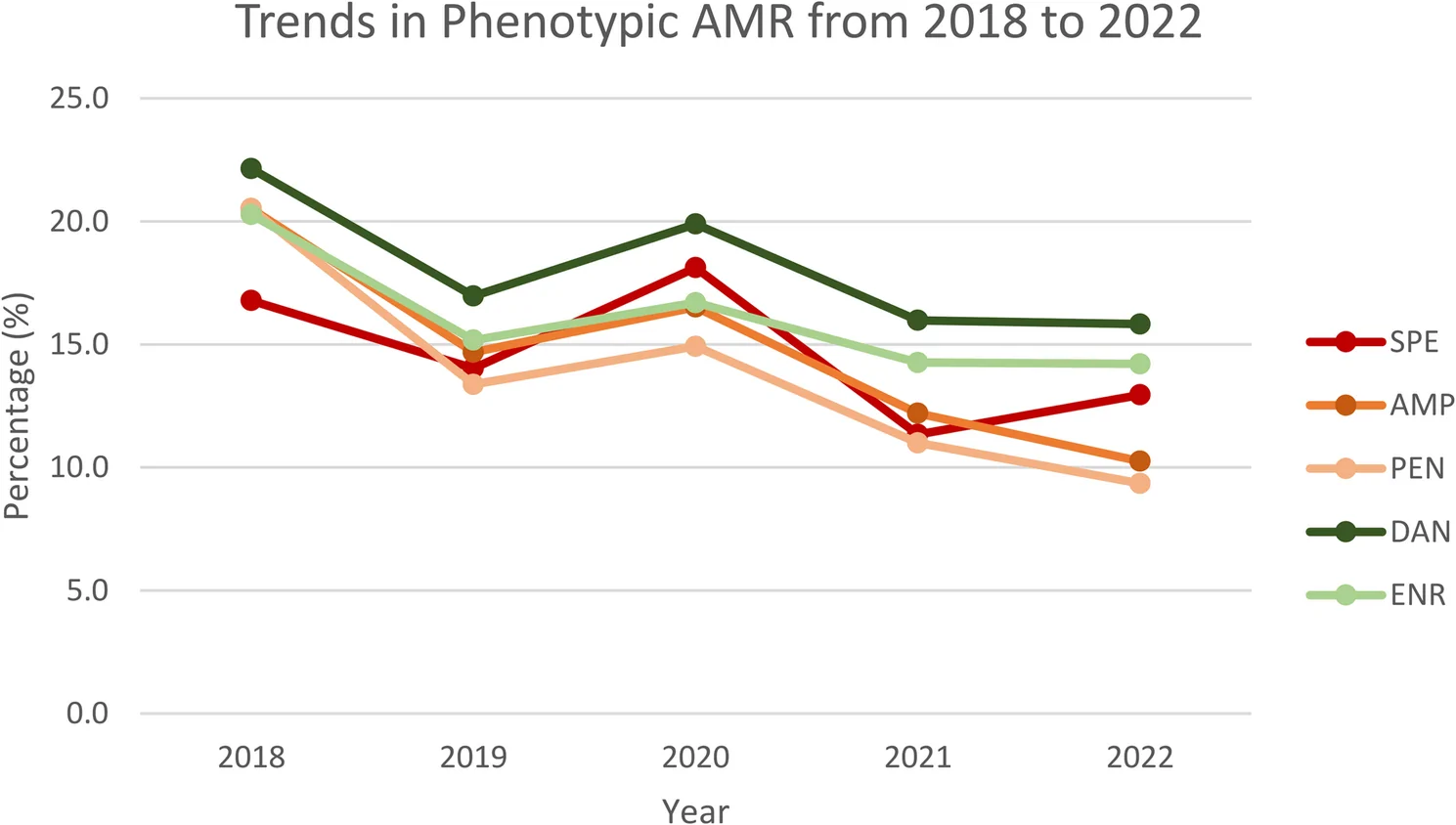
Trends in phenotypic antimicrobial resistance (AMR) from 2018 to 2022. This line graph shows the percentage of resistance (y-axis) over time (x-axis) for specific antibiotics that demonstrated a significant decreasing trend in resistance from 2018 to 2022. Antibiotics with significant reductions in resistance include spectinomycin (SPE; *p* = 0.038), ampicillin (AMP; *p* = 0.000), penicillin (PEN; *p* = 0.000), danofloxacin (DANO; *p* = 0.018), and enrofloxacin (ENRO; *p* = 0.018)

**Fig. 3:**
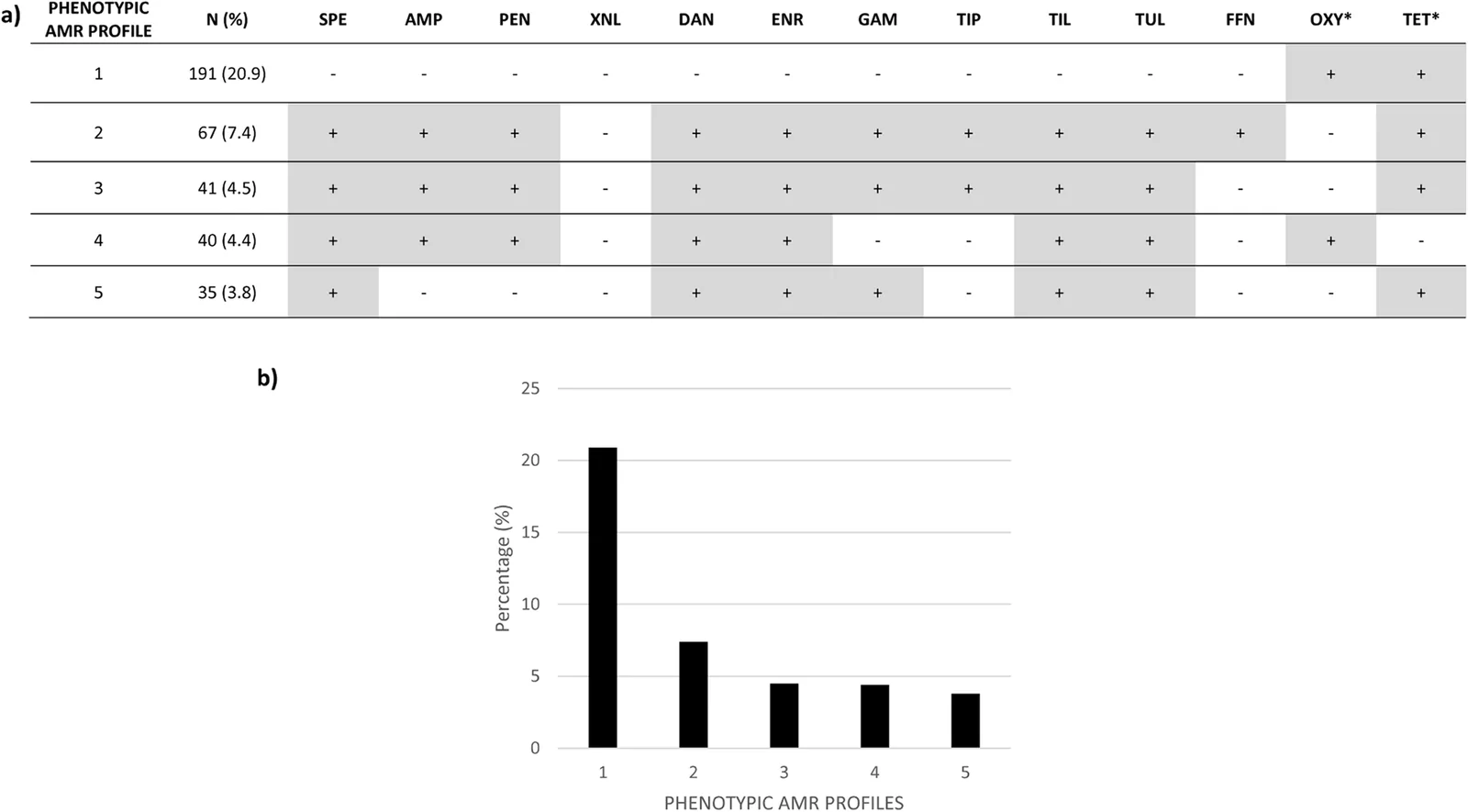
Top 5 phenotypic antimicrobial resistance (AMR) profiles and their distribution in resistant M. haemolytica *isolates (n = 913). ***a** Shows the combinations of phenotypic AMR profiles identified in this study. The plus signs (+) indicate the presence of resistance, and minus signs (−) indicate the absence of resistance. **b** Displays the percentage distribution of each phenotypic AMR profile across resistant isolates.*Oxytetracycline (OXY) and tetracycline (TET) were not tested in both Sensititre plates, thus, phenotypic AMR profile 1 is either OXY or TET

Resistance to most antimicrobials (XNL, GAM, TIP, TIL, TUL, FFN, OXY, and TET) remained stable from 2018 to 2022, with no significant changes. However, significant decreases were observed for SPE (16.8% to 12.9%, *P =* 0.038), AMP (20.5% to 9.4%, *P =* 0.000), PEN (20.5% to 10.3%, *P =* 0.000), DAN (20.3% to 14.2%, *P =* 0.018), and ENR (22.1% to 15.8%, *P =* 0.018) (Fig. 2). A total of 158 distinct AST resistance profiles were identified, excluding pan-susceptible (Supplemental file 3). Among 913 resistant isolates, the five most common phenotypic resistance profiles accounted for 374 isolates (Fig. 3). Most resistant isolates (768/913, 84.1%) exhibited TET or OXY resistance, including 193 (193/913, 21.1%) resistant to nine antimicrobials (AMP, DAN, ENR, PEN, SPE, TIP, TIL, TUL, and TET/OXY).

**Fig. 4:**
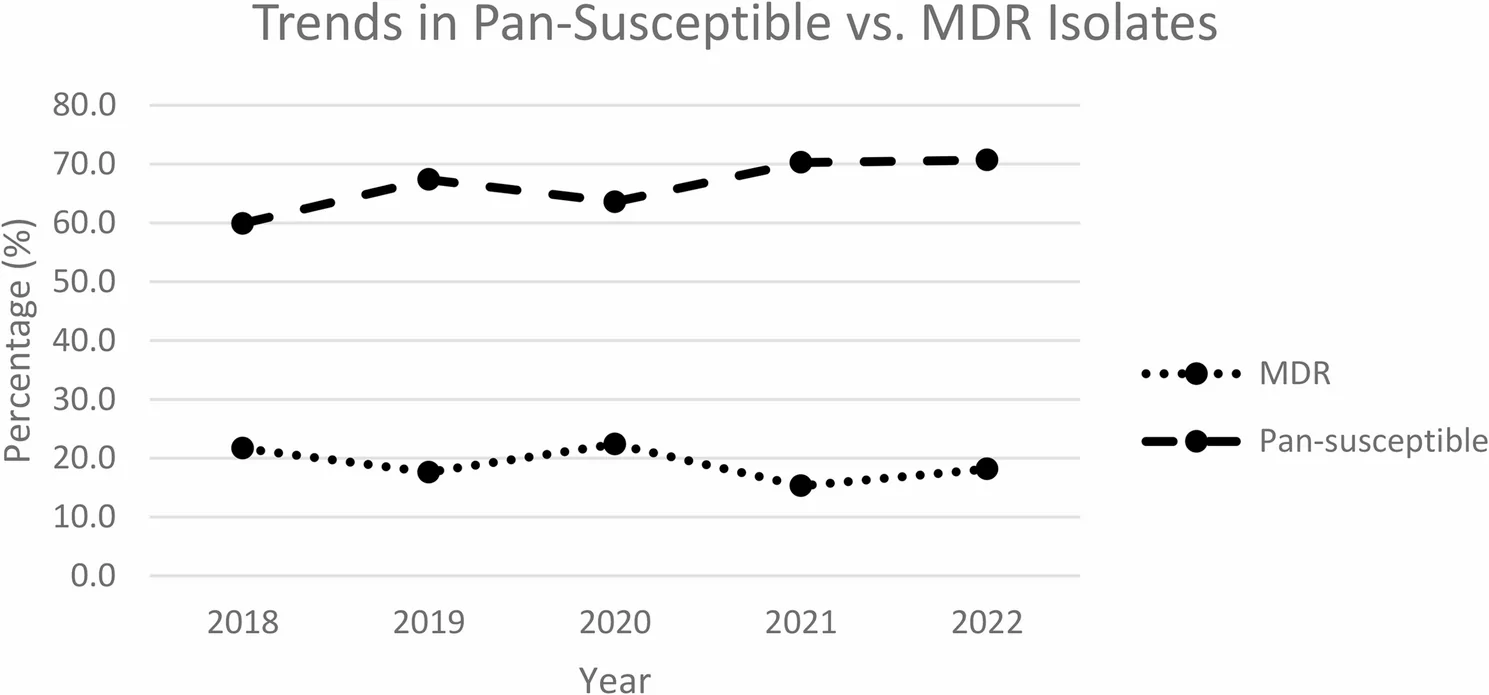
Trends in Pan-Susceptible vs. Multidrug-Resistance (MDR) Isolates Over Time: This line graph shows the percentage of pan-susceptible and MDR isolates across years. The MDR line (dotted) represents the proportion of isolates resistant to 3 or more antimicrobial classes, which showed no significant change over time. In contrast, the pan-susceptible line (dashed) illustrates a significant increasing trend in the proportion of isolates susceptible to all tested antimicrobials

Across antimicrobial classes, 43.4% (396/913) of resistant isolates were resistant to one or two classes, primarily tetracycline, often combined with a beta-lactam (penicillin subclass) or a fluoroquinolone. The remaining 56.6% (517/913) of resistant isolates were classified as MDR, representing 18.8% (517/2743) of all isolates tested. From 2018 to 2022, MDR proportions fluctuated without significant change, while pan-susceptible isolates significantly increased (59.9% to 70.7%, *P =* 0.0004) (Fig. 4).

### Genotypic antimicrobial resistance

Kraken classified all 848 sequenced isolates as *M. haemolytica* (Supplemental file 2). Of those isolates sequenced, most originated from cattle lungs (835/848, 98.5%) and just over one third contained one or more AMR genes and/or QPMs (294/848, 34.7%). In total, 18 distinct AMR genes and five QPMs were detected.

**Fig. 5:**
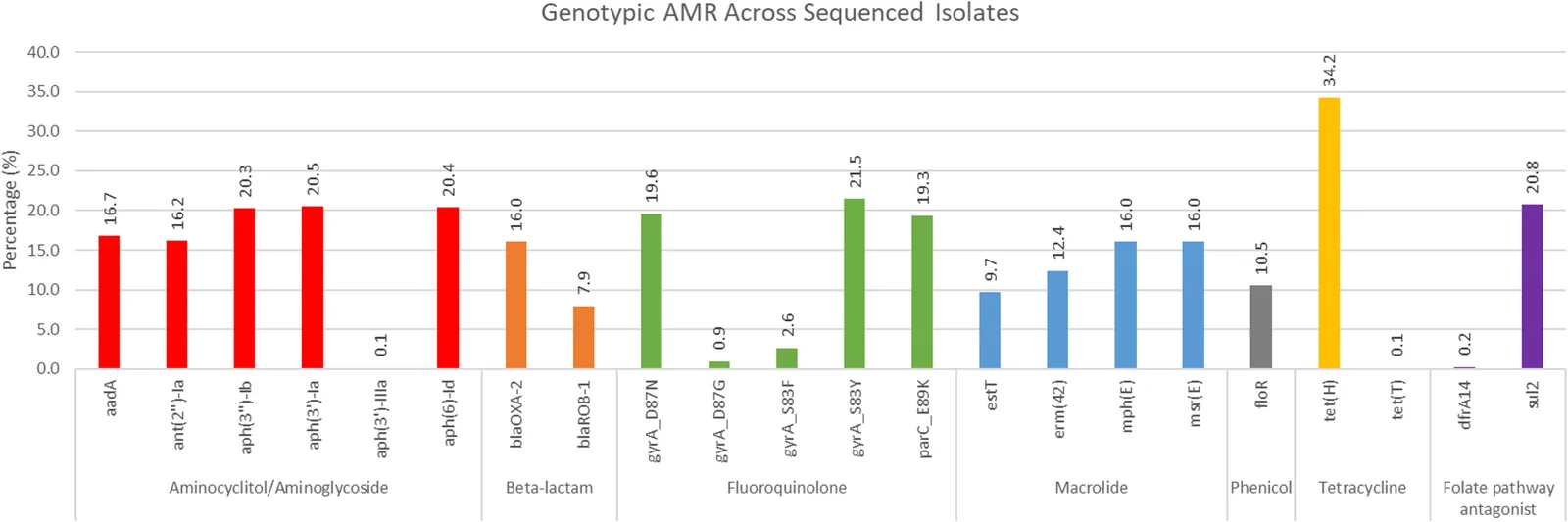
Genotypic antimicrobial resistance (AMR) across sequenced isolates (*n* = 848). Bar graph showing the percentage of isolates (y-axis) harboring various AMR genes and quinolone point mutations (QPMs) (x-axis)

AMR gene prevalence was consistent with observed phenotypic resistance. The most prevalent gene was *tet*(H) (290/848, 34.2%), consistent with the high tetracycline resistance (TET or OXY) (Fig. 5). Other AMR genes (12–21%) included macrolide resistance genes *mph*(E) (136/848, 16.0%), *msr*(E) (136/848, 16.0%), and *erm*(42) (105/848, 12.4%); aminoglycoside/aminocyclitol resistance genes *aadA* (142/848, 16.7%), *ant(2’’)-Ia* (137/848, 16.2%), *aph(3’’)-Ib* (172/848, 20.3%), *aph(3’)-Ia* (174/848, 20.5%), and *aph(6)-Id* (173/848, 20.4%); beta-lactam resistance gene *bla*_OXA−2_ (136/848, 16.0%); and sulfonamide resistance gene *sul2* (176/848, 20.8%) (Fig. 5). Less prevalent AMR genes included the phenicol resistance gene *floR* (89/848, 10.5%), macrolide resistance gene *estT* (82/848, 9.7%), and the beta-lactam resistance gene *bla*_ROB−1_ (67/848, 7.9%).

**Fig. 6:**
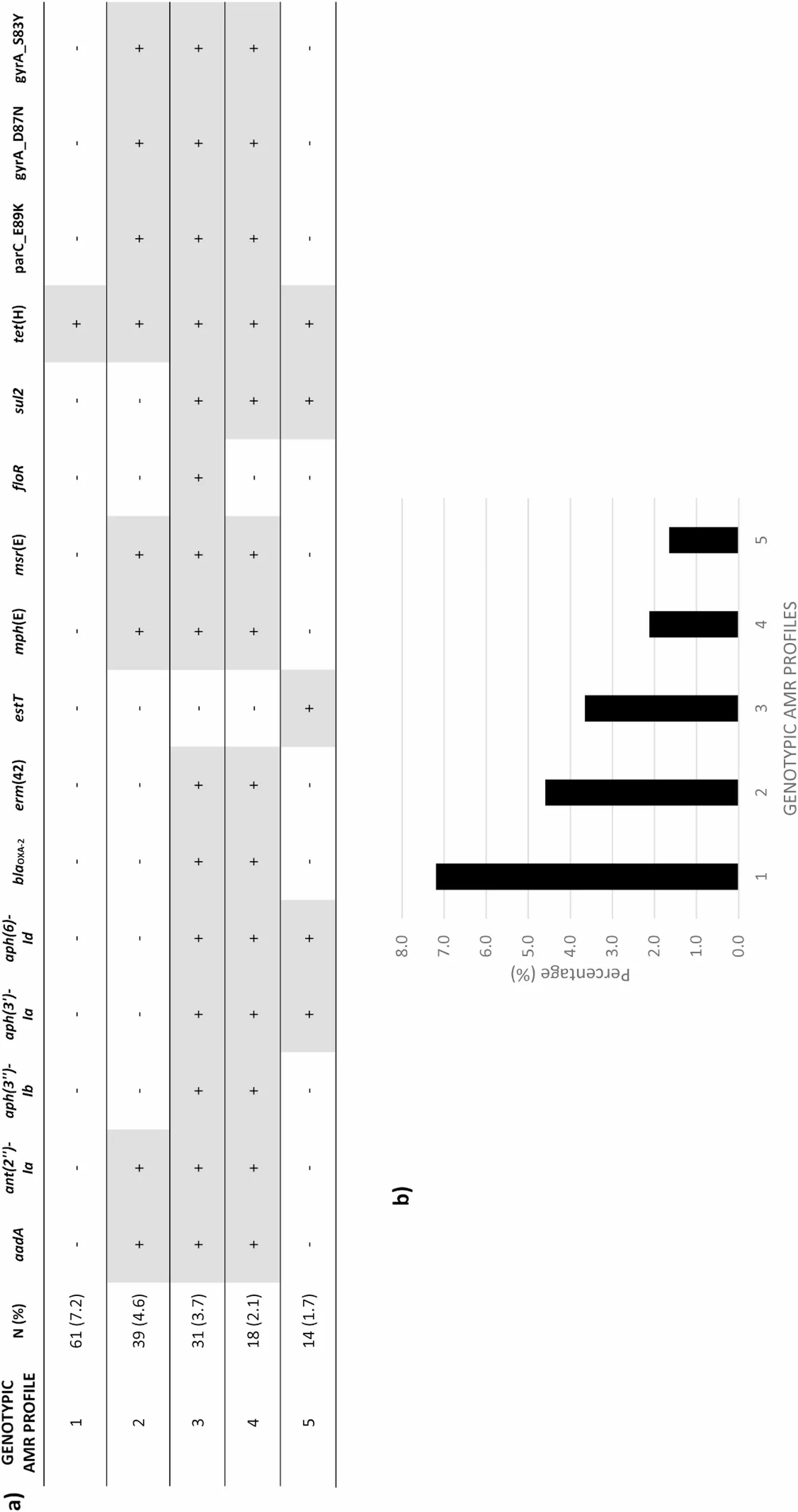
Top 5 genotypic antimicrobial resistance (AMR) profiles and their distribution in sequenced isolates (*n* = 848). **a** Shows the combinations of genotypic AMR profiles identified in this study, where plus signs (+) indicate the presence of an AMR gene and minus signs (−) indicate its absence. **b** Displays the percentage distribution of each genotypic AMR profile across the sequenced isolates

A total of 214 of 848 (25.2%) isolates had at least one QPM: parC_E89K, gyrA_D87N, gyrA_D87G, gyrA_S83F, or gyrA_S83Y. The most prevalent point mutation was gyrA_S83Y (182/848, 21.5%), followed by gyrA_D87N (166/848, 19.6%), and parC_E89K (164/848, 19.3%). Less frequent mutations included gyrA_S83F (22/848, 2.5%) and gyrA_D87G (8/848, 0.9%) (Fig. 5). Isolates with a single QPM (47/848, 5.5%) showed reduced susceptibility to both DAN (MIC = 0.5-1) and ENR (MIC = 0.5-1), with the gyrA_S83F conferring DAN resistance in eight of these isolates. Among isolates harboring two or more QPMs (167/848, 19.7%), 164 were resistant to both fluroquinolones, aligning closely with phenotypic resistance. The most prevalent combination of QPMs was parC_E89K, gyrA_D87N, and gyrA_S83Y (160/848, 18.9%) (Supplemental file 2). Overall, 60 distinct genotypic AMR profiles were identified, excluding those without AMR genes/QPMs (Supplemental file 3); the five most common profiles are summarized in Fig. 6.

**Fig. 7:**
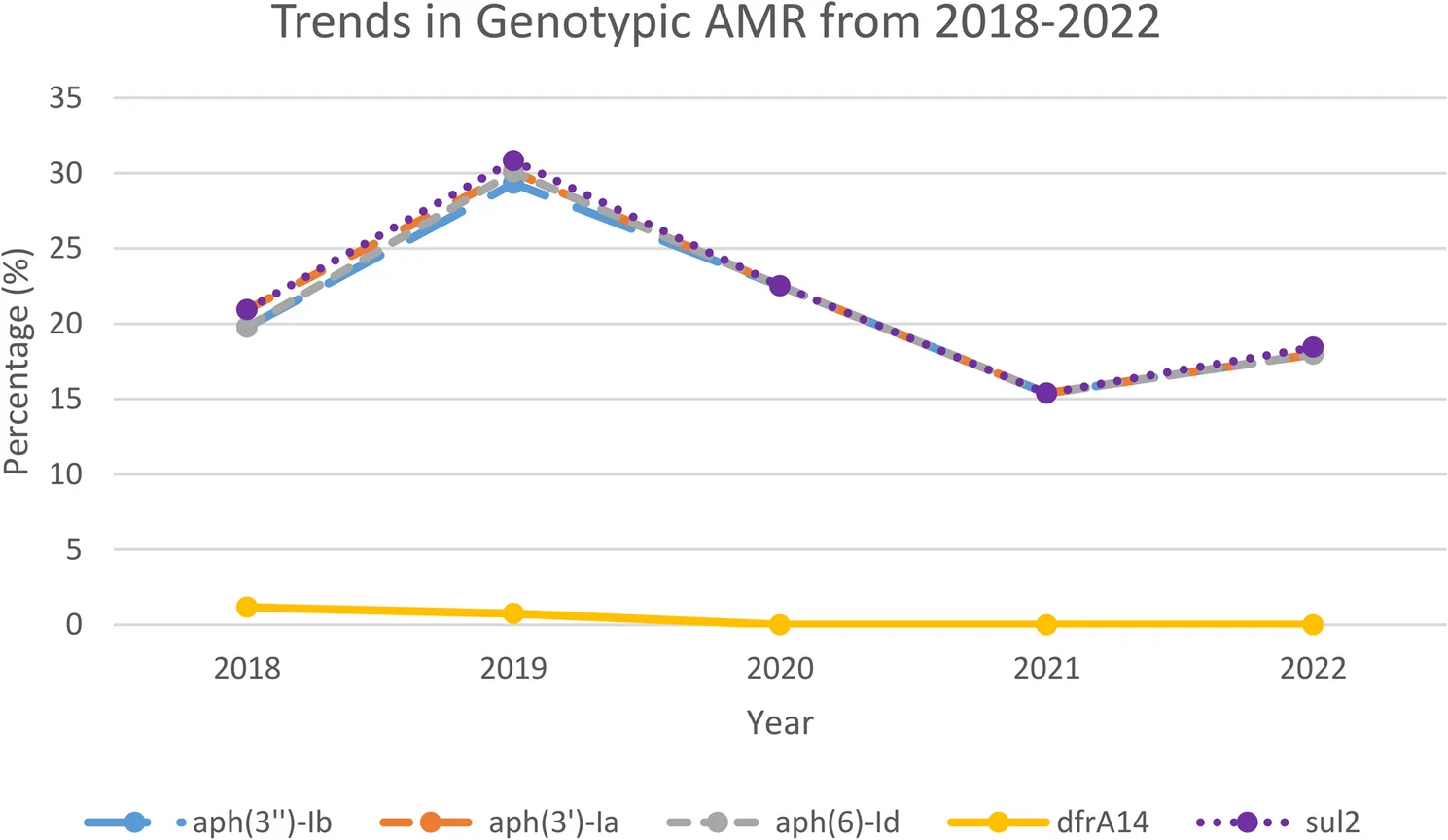
Trends in genotypic antimicrobial resistance (AMR) from 2018 to 2022. This line graph shows the percentage presence of AMR genes (y-axis) over time (x-axis) for specific AMR genes that demonstrated significant decreasing trends from 2018 to 2022. Genes showing significant decreases include aph(3’’)-Ib (*P* = 0.035), aph(3’)-Ia (*P* = 0.019), aph(6)-Id (*P* = 0.028), sul2 (*P* = 0.02), and dfrA14 (*P* = 0.037)

The prevalence of *aadA*, *ant(2”)-Ia*, *aph(3’)-IIIa*, *bla*_OXA−1_, *bla*_ROB−1_, *erm*(42), *mph*(E), *msr*(E), *floR*, *tet*(H), and *tet*(T)) and all QPMs remained unchanged from 2018 to 2022, with the exceptions of *aph(3’’)-Ib*, *aph(3’)-Ia*, *aph(6)-Id*, *dfrA14*, and *sul2* (Fig. 7). Significant decreases were observed for resistance genes *aph(3’’)-Ib* (19.8% to 18%, *P =* 0.035), *aph(3’)-Ia* (20.9% to 18%, *P =* 0.019), and *aph(6)-Id* (19.8% to 18%, *P =* 0.028), which aligns with the observed decrease in SPE resistance. The prevalence of *sul2* (20.9% to 18.4%, *P =* 0.02) and *dfrA14* (1.2% to 0%, *P =* 0.037) also significantly declined. However, we were unable to detect any changes in SDM or SXT resistance, as CLSI breakpoints are not established for these antimicrobials.

### Antimicrobial resistance genes encoded by mobile genetic elements

Of the 848 draft genomes, 732 contained a T4SS-ICE. Most (571/848, 67.3%) did not harbor AMR region 1 or 2, though 187 carried variations of AMR genes from these regions. Remaining isolates had AMR region 1 (29 isolates), AMR region 2 (76 isolates), or both (56 isolates). Sixty-five of the 105 isolates with only one AMR region also carried additional AMR genes within the T4SS-ICE, including genes typical of the opposite AMR region (Supplemental file 2).

AMR region 2 was most frequent (132/848, 15.6%) and appeared in three of the five most common genotypic AMR profiles. AMR region 1 was less frequent (85/848, 10%) but represented the third most common profile (Fig. 6). Other T4SS-ICE-positive isolates included 74 carrying only the *tet*(H) gene, and 57 with varied AMR gene combinations (Supplemental file 2). Both T4SS-associated and AMR genes were distributed throughout the length of the ICE (Supplemental Fig. 1).

Additionally, *bla*_ROB−1_ and *estT* were often co-located within a contig. Among 88 isolates with either *bla*_ROB−1_ and/or *estT*, 61 carried both genes on the same contig, six only had *bla*_ROB−1_, and 21 had only *estT*. Of these, 59 isolates with both AMR genes also harbored an IME, while 25 isolates with only one AMR gene lacked the detectable IME. IME-associated genes (*rel*, *int*) and AMR genes were distributed across the IME (Supplemental Fig. 1). All 59 IME-positive isolates also contained a T4SS-ICE, with or without AMR genes.

### Concordance between antimicrobial resistance phenotypes and genotypes

Overall, concordance between AMR phenotypes and genotypes was high (Table 4). Most antimicrobials showed > 96% concordance and almost perfect agreement (kappa > 0.87), including PEN, DAN, ENR, GAM, TIL, TUL, FFN, TET, and OXY. Slightly lower concordance (90–95%) and substantial agreement (kappa 0.71-74) were noted for SPE, AMP, and TIP (Table 4).

**Table 4:**
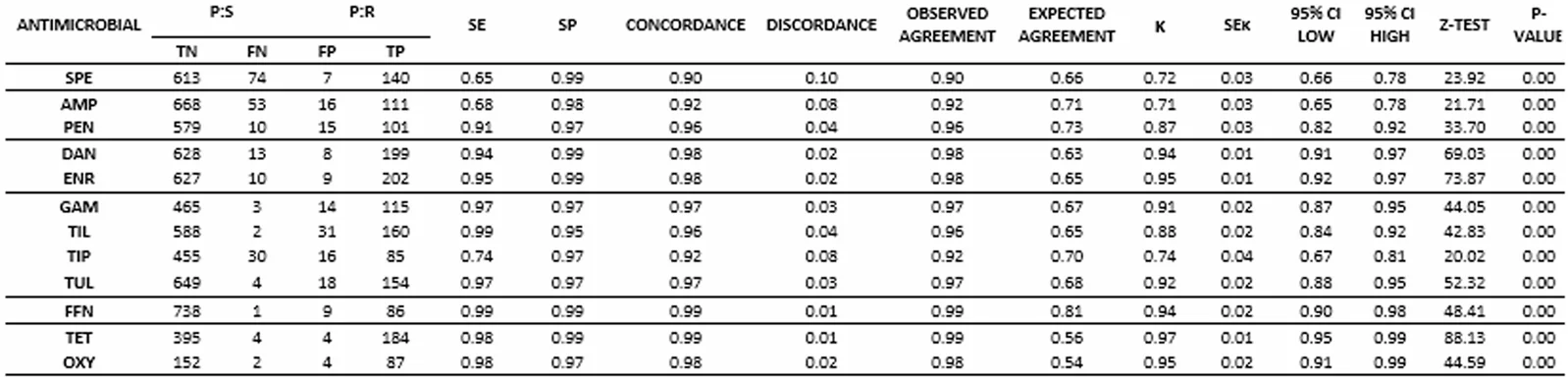
Q7

Seven isolates showed phenotypic resistance to SPE (MIC ≥ 128 µg/ml) but did not harbor any known aminoglycoside/aminocyclitol resistance genes. Conversely, three isolates were susceptible to SPE (MIC ≤ 32 µg/ml) despite carrying five resistance genes (*aadA2*, *ant(2’’)-Ia*, *aph(3’’)-Ib*, *aph(3’)-Ia*, *aph(6)-Id*). Additionally, 70 isolates were susceptible despite harboring *aph(3’’)-Ib*, *aph(3’)-Ia*, and *aph(6)-Id*, suggesting these genes alone may not confer SPE resistance. Among isolates with *bla*_OXA−2_, 52 had AMP MICs ≤ 0.25 µg/ml; however, the resistance breakpoint for AMP is ≥ 0.25 µg/mL, true MICs could not be confirmed as 0.25 µg/mL was the lowest concentration tested. Twenty isolates carried *mph*(E) and *msr*(E) but were TIP-susceptible (MIC ≤ 4 µg/ml), though all were resistant to other macrolides (GAM, TIL, TUL).

### Identification of genotypes and phylogenetic relationships

Implementation of the Clawson et al. [8, 30] SNP genotyping scheme revealed a clear divergence between the two major genotypes (Supplemental Fig. 2). Two outlier isolates (2/848) were excluded from vSNP analysis due to substantial SNP divergence and could not be accurately classified.

**Fig. 8:**
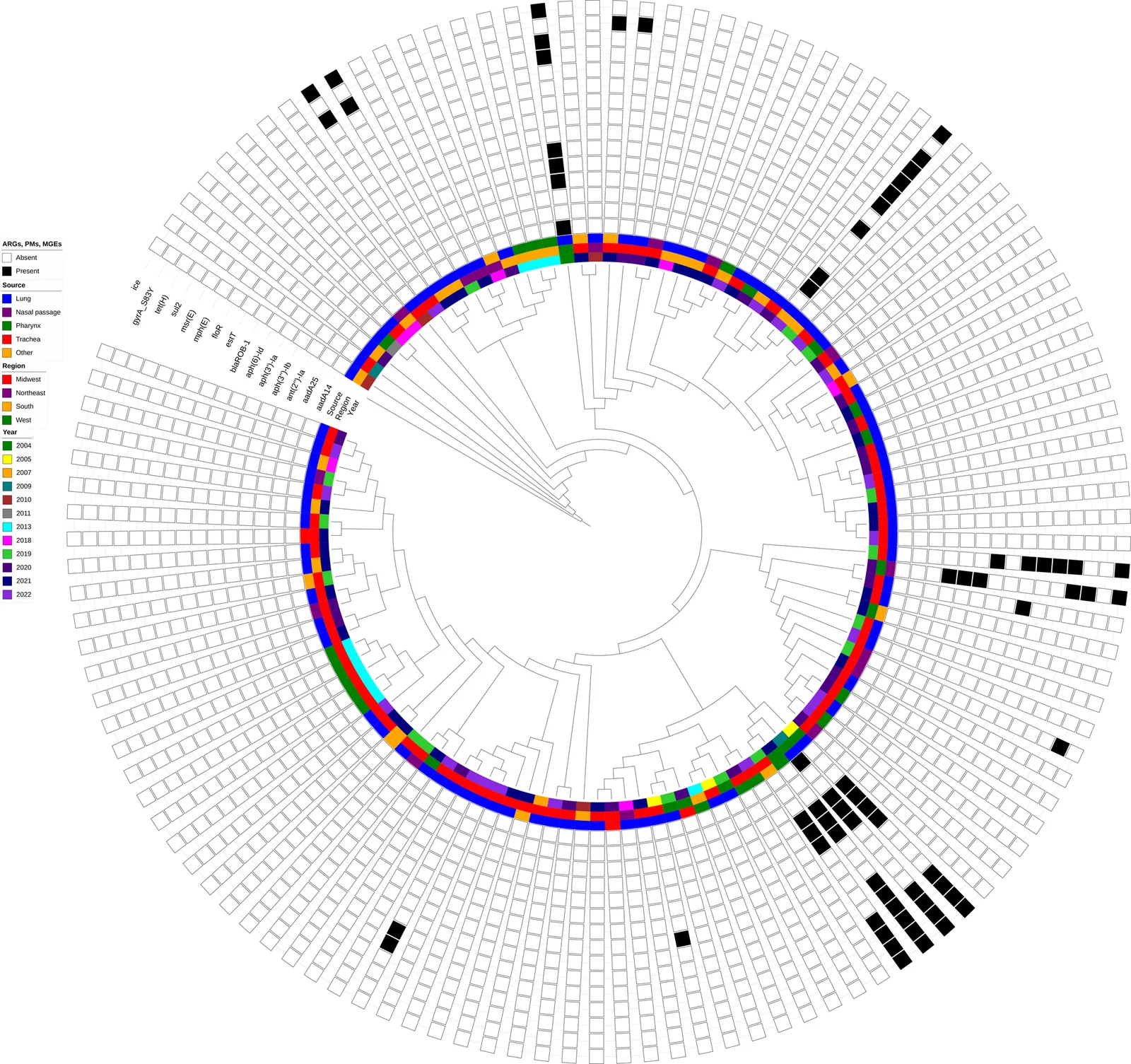
Phylogenetic tree of genotype 1 *M. haemolytica* isolates (*n* = 118), illustrating the presence and absence of antimicrobial resistance (AMR) genes, point mutations (PMs), and mobile genetic elements (MGEs). The tree also displays the source of isolation, geographical region, and the year each isolate was tested, with branch lengths ignored for visualization purposes. Tree was generated RAxML with GTR-CATI model of evolution using vSNP

Among 846 analyzed isolates, 11.5% (97/846) belonged to genotype 1 (Supplemental Fig. 2). Most genotype 1 isolates (85/97, 87.6%) lacked AMR genes, QPMs, and MGEs; three only had the gyrA_S83Y QPM. Nine isolates harbored AMR genes, seven within T4SS-ICE, and two also had the gyrA_S83Y QPM. Clustering was observed among isolates with QPMs, AMR genes, and MGEs, particularly those harboring T4SS-ICE and AMR genes *aph(3”)-Ib*, *aph(3’)-Ia*, *aph(6)-Id*, *sul2*, and *tet*(H) (Fig. 8). In contrast, genotype 2 was predominant (Supplemental Fig. 2), accounting for 88.5% (749/846). The majority (724/749, 96.7%) contained a T4SS-ICE, and 37.9% (284/749) harbored AMR genes within the T4SS-ICE. Of these, 161 carried AMR regions 1 and/or 2, and 59 also contained an IME with *bla*_ROB−1_ and *estT*.

**Fig. 9:**
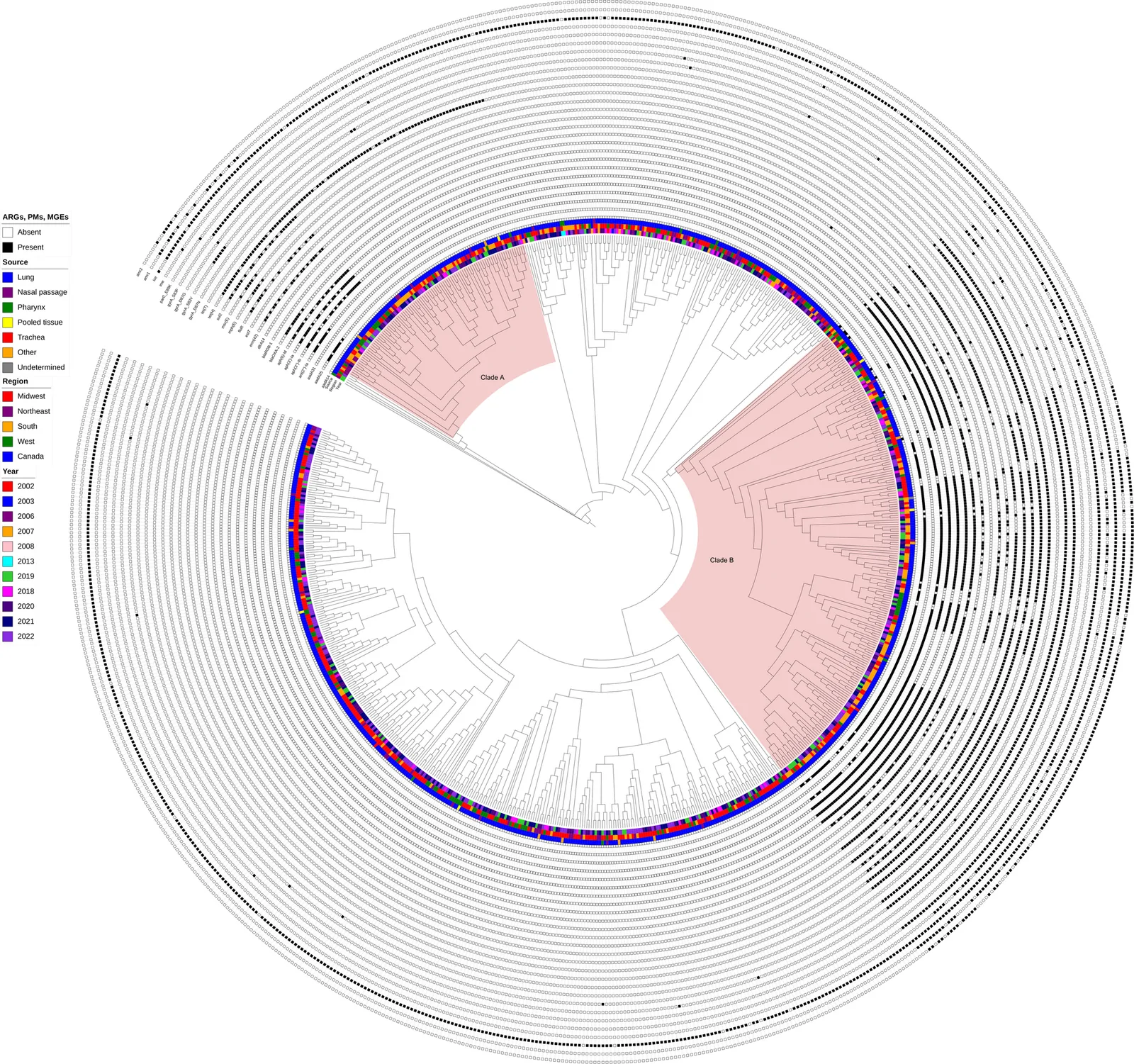
Phylogenetic tree of genotype 2 *M. haemolytica* isolates (*n* = 801), illustrating the presence and absence of antimicrobial resistance (AMR) genes, point mutations (PMs), and mobile genetic elements (MGEs). The tree also highlights the source of isolation, geographical region, and the year each isolate was tested, with branch lengths ignored for visualization purposes. Unique clades A and B, which contain AMR genes, are distinguished in pink. Tree was generated RAxML with GTR-CATI model of evolution using vSNP

**Fig. 10:**
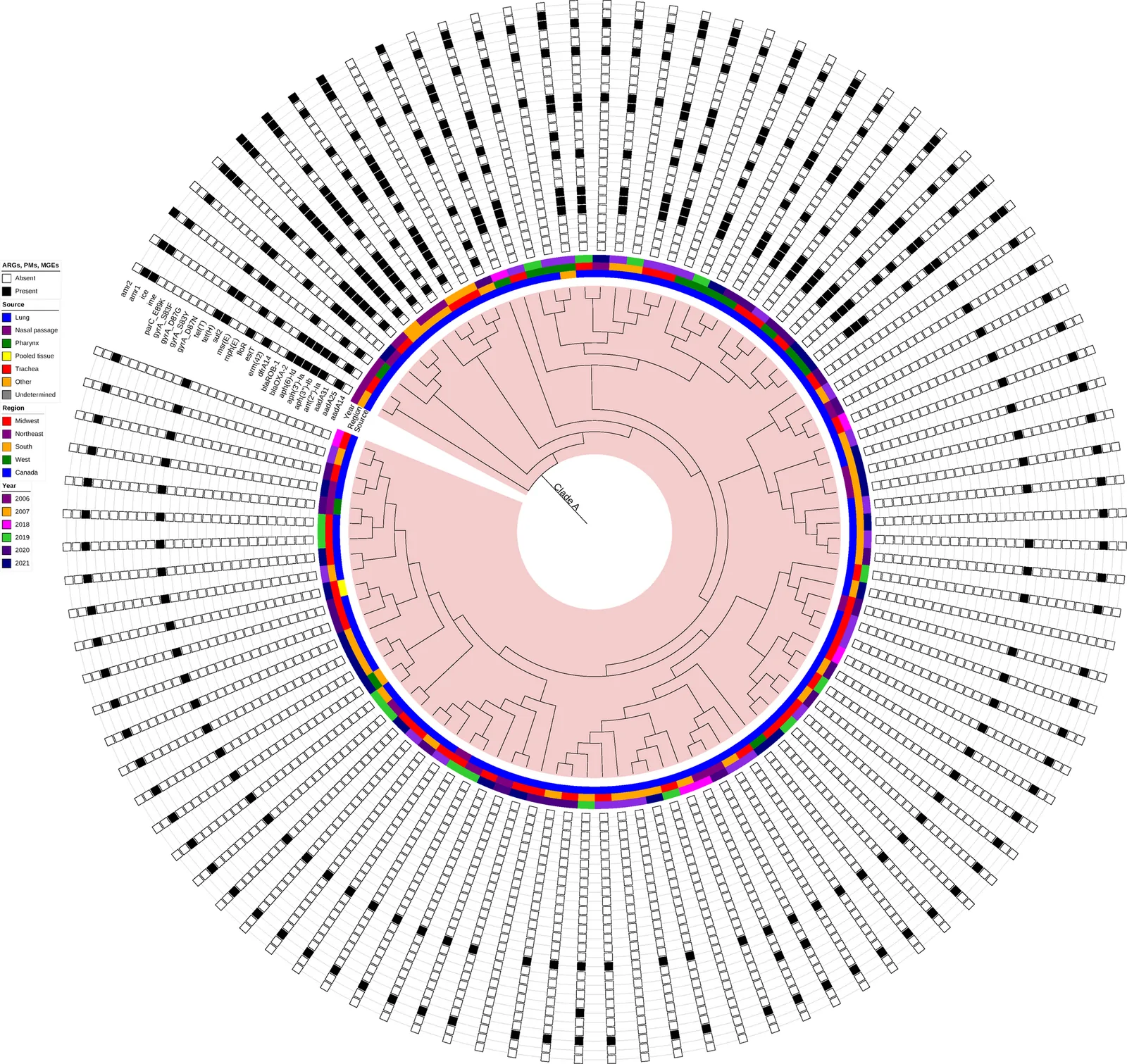
Phylogenetic tree of *M. haemolytica* Genotype 2 Clade A isolates (*n* = 89), depicting the presence or absence of antimicrobial resistance (AMR) genes, point mutations (PMs), and mobile genetic elements (MGEs). The tree also highlights the source of isolation, geographical region, and the year each isolate was tested, with branch lengths ignored for visualization purposes. Tree was generated RAxML with GTR-CATI model of evolution using vSNP

**Fig. 11:**
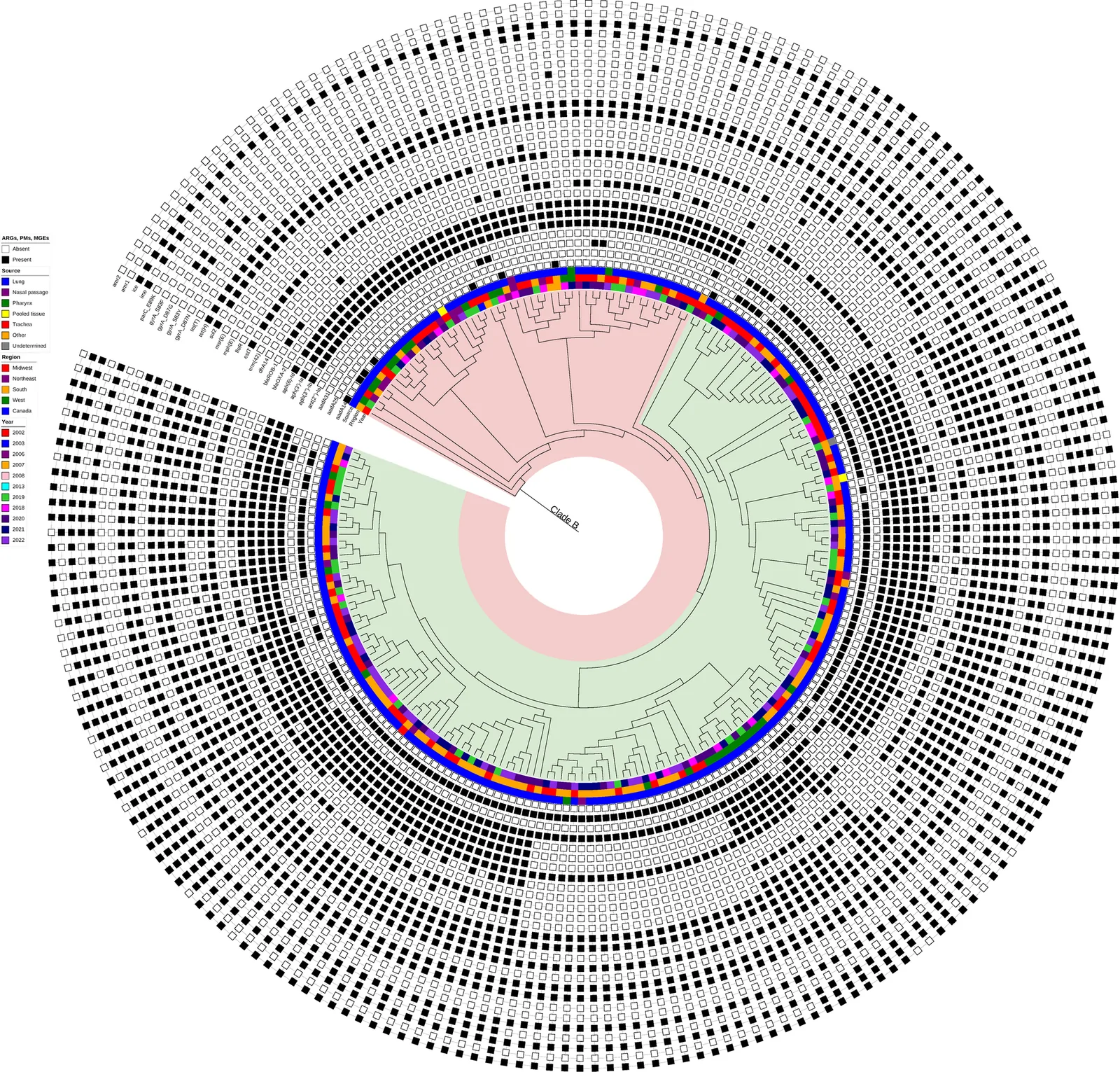
Phylogenetic tree of *M. haemolytica* Genotype 2 Clade B isolates (*n* = 215), depicting the presence or absence of antimicrobial resistance (AMR) genes, point mutations (PMs), and mobile genetic elements (MGEs). The tree also highlights the source of isolation, geographical region, and the year each isolate was tested, with branch lengths ignored for visualization purposes. Subclade of isolates possessing the QPM combination gyrA_D87N, gyrA_S83Y, and parC_E89K (*n* = 160) is highlighted in green. Tree was generated RAxML with GTR-CATI model of evolution using vSNP

Phylogenetic analysis of 298 genotype 2 isolates carrying AMR genes (this study and Clawson et al. [8]) revealed two distinct clades (Fig. 9). Clade A included 89 isolates from this study and nine from Clawson; most (59/89) harbored only *tet*(H), while 18 carried additional AMR genes, including *aph(3”)-Ib*, *aph(3’)-Ia*, *aph(6)-Id*, *sul2*, and the gyrA_S83F QPM (Fig. 10). Clade B contained 215 isolates (200 from this study, 15 from Clawson) and included a subclade where 160 isolates possessed the QPM combination gyrA_D87N, gyrA_S83Y, and parC_E89K; most (151/160, 94.4%) also contained AMR regions 1 and/or 2 (Fig. 11).This subclade encompassed the second-, third-, and fourth-most common genotypic AMR profiles. In contrast, Clade A showed only single QPMs, and gyr_D87N was absent.

Overall, isolates with similar genotypic AMR profiles (Fig. 6) clustered together. Across both genotype 1 and 2 trees, isolates appear randomly distributed across years, showing no evidence of temporal clustering. Some clustering was observed based on the source of isolation, though this may reflect sampling bias, as most isolates were sourced from cattle lungs, while isolates from other sources were randomly distributed. Additionally, isolates from the same geographic region, particularly the Midwest and South, tended to cluster, suggesting close relatedness among isolates from these regions.

## Discussion

This study provides a comprehensive analysis of AMR in *M. haemolytica* isolates from clinical diagnostic cases submitted over a five-year period, offering insights into the AMR profiles and the prevalence of genotype 2 in this important cattle pathogen in the U.S.

### Antimicrobial resistance prevalence in clinically isolated *M. haemolytica*

Between 2018 to 2022, we observed a 33% prevalence of AMR in isolates primarily recovered from clinical lung samples in sick cattle. A Canadian longitudinal study similarly reported a 37% AMR prevalence in nasopharyngeal isolates from cattle diagnosed with BRD [54]. In contrast, *M. haemolytica* from lung tissue of BRD mortalities in North American feedlots (55 isolates from U.S. and Canadian feedlots) showed 72% resistance, more than double our findings [26]. Differences in resistance rates may be due to sample sizes and potential geographical differences in antimicrobial use, management practices, and sample methods between regions. Despite these variations, AMR prevalence values underscore the widespread presence of resistance in *M. haemolytica*. Importantly, antimicrobial use in healthy animals, whether through metaphylaxis or environmental exposure, may drive resistance development, and resistant strains can spread within herds regardless of health status, reinforcing the potential for widespread transmission.

Tetracycline resistance was the most prevalent, with nearly 32% (271/848) of isolates resistant to tetracycline or oxytetracycline, mediated by the *tet*(H) gene, aligning with previous research [8, 50, 55]. Historically, tetracyclines were widely used in cattle for growth promotion and disease prevention [56, 57] until these practices were banned by the FDA in 2017 (FDA 2013). The exact drivers of tetracycline resistance in *M. haemolytica* remains unclear.

Macrolide and fluoroquinolone resistance were the second most common (~ 20%) in *M. haemolytica*, particularly against tulathromycin, tilmicosin, danofloxacin, and enrofloxacin, consistent with previous studies in cattle with BRD [32, 46, 48, 58]. Macrolides remain a first-line treatment for BRD due to their efficacy against *M. haemolytica* and *P. multocida* and ability to achieve therapeutic levels in the lungs of cattle, while fluroquinolones are used as second-line therapy for severe BRD cases [12, 59]. Macrolide resistance was primarily associated with *mph*(E) (136/848, 16.0%) and *msr*(E) (136/848, 16.0%), followed by *erm*(42) (105/848, 12.4%) and *estT* (82/848, 9.7%), which have been commonly detected in other studies [17, 19, 21, 23, 50, 55, 58]. However, tilmicosin resistance was slightly higher (543/2743, 19.8%), which may be attributed to the presence of *erm*(42) and/or *estT*.

Unlike AMR genes located in MGEs, QPMs contribute to quinolone resistance through a stepwise selection of mutations in chromosomal genes (*gyrAB* and *parCE*), leading to increased MICs to quinolones and ultimately resistance [52, 60–62]. Reduced susceptibility and fluoroquinolone resistance in *M. haemolytica* has been linked to QPMs in *gyrA* (S83F, S83Y, D87N, and D87G) and *parC* (E89K and S85I) as shown in our study [22, 46, 48, 55]. The most prevalent QPM in our study was gyrA_S83Y, often combined with parC_E89K and gyrA_D87N (19.0%). This contrasts with previous reports where gyrA_S83F [10, 23, 46, 55, 63] and the combination of parC_E89K, gyrA_D87N, and gyrA_S83F was more common [55], suggesting possible geographic or temporal differences in selective pressure, strain variation, and mutation fitness [52].

Florfenicol, a phenicol antimicrobial and another key first-line treatment for BRD [12, 64], showed a lower resistance rate of 10% (273/2743) in our study, much lower than the 31% (35/114) reported by Wickware et al. [55]. Resistance was primarily associated with the presence of the *floR* gene [8, 32, 55, 59]. Spectinomycin, an aminocyclitol occasionally used when other options are unavailable [12, 64], showed a resistance rate of 14.5% (398/2743), consistent with previous findings [21, 23, 50]. Spectinomycin resistance was associated with multiple aminocyclitol/aminoglycoside resistance genes, often in combination: *aadA* (16.7%), *ant(2’’)-Ia* (16.2%), *aph(3’’)-Ib* (20.3%), *aph(3’)-Ia* (20.5%), and *aph(6)-Id* (20.5%), a result supported in previous *M. haemolytica* studies [8, 10, 55].

Beta-lactams, such as penicillin and ampicillin, are less commonly used antimicrobials for BRD due to limited efficacy against BRD-associated pathogens [12, 64]. Approximately 15% of *M. haemolytica* isolates were resistant to one or both beta-lactam drugs, as has been previously observed, this was primarily mediated by *bla*_OXA−2_ (16.0%) and, to a lesser extent, *bla*_ROB−1_ (7.9%) [22, 23, 54, 59]. This pattern may reflect the use of these antimicrobials to treat concurrent infections in animals that also carried *M. haemolytica*, thereby exposing the organism to selective pressure without it being the primary target of treatment [65, 66]. Resistance to ceftiofur remains rare (< 1%) even in ceftiofur-treated cattle [21, 22, 24, 55, 67], including our study (0.1%). The single ceftiofur resistant isolate in our study lacked known resistance genes, further supporting the absence of extended-spectrum beta-lactamase genes in *M. haemolytica* [68]. This is encouraging, as ceftiofur is the only broad-spectrum cephalosporin approved in the U.S. for treatment of cattle and is often used when first- or second-line treatments fail [64, 68].

Overall, 18.8% (517/2743) of *M. haemolytica* isolates were MDR, consistent with other studies and highlighting the challenge MDR strains pose in treating severe or chronic BRD cases [11, 20, 22, 50, 63]. Importantly, previous studies have shown a strong association between MDR *M. haemolytica* and cattle treated with antimicrobials, either metaphylactically or therapeutically, compared to healthy cattle [6, 11, 24], and is primarily driven by T4SS-ICEs carrying multiple AMR genes.

Despite the widespread resistance to several antimicrobial classes, most isolates (1830/2743, 66.7%) remained pan-susceptible, aligning with observations by Noyes et al. [6], who reported similar findings in cattle at feedlot arrival and later during the feeding period. This suggests that, while AMR is a concern, a substantial proportion of *M. haemolytica* isolates remain susceptible to commonly used antibiotics, providing hope for continued effective treatment options.

### Concordance between phenotypic and genotypic resistance

A major strength of this study is the evaluation of concordance between phenotypic and genotypic resistance across 848 isolates. Several studies have demonstrated the ability to predict AMR using WGS data on AMR genes and mutations, often with a high genotype-phenotype concordance [50, 55, 60, 69, 70].

Our findings demonstrate strong concordance (> 96%) and strong agreement (kappa > 0.87) between phenotypic resistance and presence of AMR determinants for most antimicrobials. Similarly, Wickware et al. reported high concordance (> 93%) for multiple antimicrobials in *M. haemolytica*, including danofloxacin, enrofloxacin, tilmicosin, tildipirosin, tulathromycin, florfenicol, and tetracycline [55]. In their study, florfenicol and tilmicosin showed the lowest concordance (94 and 93%, respectively), whereas our study reported higher concordance (99% and 96%). Conversely, Wickware et al. [55] observed greater concordance for tildipirosin (96%) compared to ours (92%). Concordance for tildipirosin, ampicillin, and spectinomycin in our dataset was slightly lower than for other antimicrobials (90–92%). Potential explanations include the presence of novel or undetected AMR genes, fragmented genome assemblies that obscure AMR determinants, or silent AMR genes that are expressed only under antimicrobial pressure. Additionally, differences between the two studies may reflect sample size variation, as our much larger dataset likely improved sensitivity and specificity. It is also important to note that Wickware et al. [55] sourced isolates from three veterinary diagnostic laboratories participating in the NAHLN AMR Pilot Project. However, due to the absence of shared identifiers, we could not determine whether any isolate overlap existed between datasets, limiting direct comparability.

Given our large sample size of sequenced isolates, our study contributes to the growing evidence that AMR genes are reliable indicators of phenotypic resistance in *M. haemolytica*. These findings support WGS as a robust tool for AMR surveillance and highlights its potential as a complementary method to AST. Beyond surveillance, WGS could serve as a diagnostic tool for detecting resistance and guiding treatment decisions in BRD clinical cases.

### Temporal trends of resistance

In contrast to our study, Portis et al. [17] and Holschbach et al. [21] reported increases in resistance to danofloxacin, enrofloxacin, florfenicol, tilmicosin, and tulathromycin in *M. haemolytica*. However, while these studies covered 10‑year periods (2000–2009 and 2008–2017, respectively), the decline in reduced susceptibility or resistance to fluoroquinolones observed in our study may indicate a downward trend occurred after 2018. While no changes were observed in penicillin resistance from 2000 to 2009, both the current study and Holschbach et al. [21] observed a decrease in resistance to penicillin and/or ampicillin, suggesting a decline in resistance to these beta-lactams starting around 2008. Resistance to florfenicol, tilmicosin, and tulathromycin fluctuated, decreasing from 2000 to 2009, increasing 2008 to 2017, and stabilizing between 2018 and 2020.

While differences in temporal trends across studies highlight a potential shift in resistance patterns, it is important to consider geographic variation. Portis et al. collected 2977 *M. haemolytica* isolates from 24 U.S. and Canadian veterinary diagnostic laboratories [17], whereas Holschbach et al. [21] examined > 4000 isolates primarily from Wisconsin (> 90%). Regional antimicrobial use, management practices, and *M. haemolytica* exposure likely influence observed patterns, underscoring the need for cautious interpretation. Importantly, these earlier studies relied solely on phenotypic data and did not include genotypic information, such as QPMs or AMR gene profiles, which limits the ability to determine whether temporal changes in resistance reflect true shifts in susceptibility or changes in the prevalence of specific clonal lineages. Incorporating QPM and broader AMR gene data would provide clearer insight into the evolutionary dynamics underlying fluoroquinolone resistance and help distinguish whether observed trends are driven by reduced antimicrobial pressure or clonal expansion of particular *M. haemolytica* lineages, or horizontal dissemination of AMR elements.

Although *M. haemolytica* remains resistant to several antimicrobial classes critical for cattle treatment, the observed increase in pan-susceptibility and decline in resistance may reflect changes in veterinary practices, including reduced antimicrobial use or improved antimicrobial stewardship. These trends align with ongoing efforts for the prudent use of antimicrobials in animals to combat AMR, as outlined in health initiatives like the U.S. National Action Plan [71] and the World Health Organization’s Global Action on AMR [72]. Despite these efforts, the prevalence of resistance in *M. haemolytica* underscores the need for continued surveillance to track resistance trends and inform effective antimicrobial stewardship. Future research should examine antimicrobial use in treating sick cattle or in farm-based metaphylaxis during AMR surveillance to establish clearer correlations between antimicrobial use and AMR patterns. WGS provides a significant advantage in AMR surveillance by identifying current trends and emerging resistance mechanisms. Our study is unique in combining phenotypic AMR trends with genotypic data (AMR genes and QPMs), providing insight into genotypic AMR changes in the *M. haemolytica* population in sick cattle over time.

### Genotype 2 *M. haemolytica* and associated mobile genetic elements

A SNP-based genotyping scheme developed by Clawson et al. identified two major *M. haemolytica* genotypes: genotype 1, typically associated with non-clinical nasopharyngeal isolates, and genotype 2, predominantly linked to clinical lung isolates from cattle with BRD [8]. Clawson et al. further demonstrated that genotype 2 harbors ICE homologs of ICE*Pmu1* and ICE*Mh1*, with subtype 2b frequently associated with AMR genes [8]. Our findings support these observations, as 88.5% (749/846) of sequenced isolates were genotype 2, most originating from the lungs of sick cattle. Nearly all genotype 2 isolates (724/749, 96.7%) contained T4SS-ICE homologs, a feature that clearly distinguishes genotype 2 from genotype 1, as reflected in phylogenetic analyses.

Among isolates carrying at least one AMR gene, 96.6% (284/294) were genotype 2. More than half of these (161/284, 56.7%) contained AMR regions 1 and/or 2, with region 2 being most prevalent. These AMR regions frequently co-occurred on the same contig and were integrated within T4SS-ICE homologs, consistent with previous studies [8, 23, 27, 28]. However, resistant isolates exhibited diverse AMR gene combinations within T4SS-ICEs, indicating variability in AMR carriage [8, 27]. Genotype 2 isolates carrying AMR genes clustered into two unique clades, both related to genotype 2b isolates from the Clawson et al. study, which were associated with AMR genes encoded by ICE*Pmu1* and ICE*Mh1* homologs in lung clinical isolates [8]. Furthermore, isolates with similar QPM patterns clustered within the same unique clades as genotype 2 isolates carrying AMR genes, suggesting a potential link between QPM patterns and the evolutionary path of these isolates.

Consistent with Clawson et al. (5/150, 3.3%), genotype 1 was less prevalent than genotype 2 in our study (97/846, 11.5%) among lung isolates from cattle with respiratory disease. Furthermore, while most of the *M. haemolytica* isolates from our study contained a T4SS-ICE, 440 of those isolates did not harbor any AMR genes, aligning with previous findings that non-AMR ICEs are common in *Pasteurellaceae* [8, 23, 43]. Beyond AMR genes, T4SS-ICEs also carry cargo genes encoding virulence and metabolic factors, enhancing bacterial fitness and host adaptation [61, 62]. The strong association of T4SS-ICEs with genotype 2 suggests these MGEs may not only facilitate AMR dissemination but also play a role in the pathogenicity of *M. haemolytica* in BRD [73–75]. Since AMR does not directly correlate with virulence, future studies should identify which ICE-associated factors drive genotype 2’s dominance in diseased cattle.

Additionally, we discovered an IME carrying *bla*_ROB−1_ and *estT* in 59 genotype 2 isolates. Full coverage of IME-associated genes (*rel*, *int*, and *mbeC*) along with AMR genes (*bla*_ROB−1_ and *estT* ) within the IME structure indicated co-localization and potential mobility [76, 77]. Interestingly, ICEFinder identified the macrolide esterase gene *estT*, a gene recently discovered in 2023 [78], as *rdmC*, which encodes an aclacinomycin methyl esterase [79]. In contrast, Deschner found *estT* within an ICE*Mh1* homolog alongside other AMR genes [79], while other studies reported *bla*_ROB−1_ on plasmids in BRD pathogens [80, 81]. These findings highlight the accessibility of these genes across MGEs, facilitating their horizontal transfer. The presence of the IMEs harboring *bla*_ROB−1_ and *estT* in genotype 2 clinical lung isolates underscores their role in AMR dissemination within BRD-associated populations, contributing to difficult-to-treat infections in cattle, driven by the co-selection of AMR genes and/or MDR [20, 64].

A key difference between this study and Clawson et al. is that genotype 1 isolates in our study were recovered more frequently from lung samples (69/97, 71.1%) compared to 14.7% (5/34) in their study, despite genotype 1’s typical association with non-clinical nasopharyngeal samples [8]. Conversely, a small proportion of genotype 2 isolates (46/749, 6.1%) in our study originated from non-lung sources (nasal passage, pharynx, trachea, and pooled tissue). Since our dataset is composed of clinical samples, future studies incorporating healthy, sick, and deceased cattle would provide a more comprehensive understanding of genotype 1 across diverse populations.

## Limitations and future directions

While the large sample size and extended time frame provided a comprehensive overview of AMR trends in *M. haemolytica*, this study may be subject to bias due to the nature of the collected samples. Isolates submitted to veterinary diagnostic laboratories are often collected after antimicrobial treatment failure, which increases the selection pressure and promotes the emergence of AMR bacteria in BRD cases [20, 24]. Consequently, the sample population may overrepresent cases with poor therapeutic outcomes, limiting its ability to reflect AMR levels in the broader cattle population. Additional limitations include the inability to use state-level origin data due to confidentiality restrictions and the lack of antimicrobial treatment histories for these diagnostic cases. Future research could incorporate treatment records/antimicrobial use and farm type (dairy or beef), as these factors could clarify links between treatment practices and AMR development.

Moreover, such information would provide valuable insights into the distribution of AMR and MGE in genotype 2 *M. haemolytica*, particularly within a more representative population that includes healthy, sick, and deceased cattle. Although thousands of *M. haemolytica* genomes are publicly available, critical metadata such as AST phenotypes, isolation source, source type, and geographical location are often missing, limiting efforts to identify potential drivers of AMR and the evolution of genotype 2 *M. haemolytica*. Additionally, since our isolates originated from multiple veterinary diagnostic laboratories, variations in sample collection, processing, and procedures (e.g., DNA isolation and WGS) across laboratories may introduce further variability.

## Conclusions

This study provides valuable insights into the AMR profiles of *M. haemolytica* isolates from BRD cases, showing both prevalence of resistance and strong genotype-phenotype concordance. Our findings emphasize the significant role of MGEs, particularly T4SS-ICEs and IMEs, in the dissemination of resistance within the genotype 2 *M. haemolytica* population. Despite AMR remaining a persistent challenge, we observed promising trends over a five-year period, with a decrease in resistance to commonly used antimicrobials for treating BRD and associated pathogens. These trends underscore the potential impact of antimicrobial stewardship measures. Moving forward, continued surveillance of genotype 2 *M. haemolytica* and AMR is critical to inform treatment strategies, optimize antimicrobial use, and mitigate the spread of genotype 2 *M. haemolytica* and AMR in cattle. This study also highlights the importance of WGS as an important tool in national AMR surveillance efforts, enhancing our ability to track and understand the spread of AMR at a broader scale.

## Supplementary Information

Supplementary Material 1: Supplemental file 1. Metadata, AMR Genes, Susceptibility Results, and NCBI Project Details for all sequences used.

Supplementary Material 2: Supplemental file 2. Metadata and Susceptibility Results for all NAHLN M. haemolytica isolates.

Supplementary Material 3: Supplemental file 3. Phenotypic and Genotypic AMR Profiles.

Supplementary Material 4: Supplemental Fig. 1. Comparison of MGE, ICE*Mh1*, ICEP*mu1*, and IME Structures in *M. haemolytica* Strains. The structural comparison of MGEs—ICE*Mh1*, ICEP*mu1*, and IME—from *M. haemolytica* strains was determined using WGS. ICE*Mh1* (NCBI accession no. CP005383) and ICEP*mu1* (NCBI accession no. CP003022), while the IME originated from an isolate in our collection (NCBI accession no. JBNXNN000000000). Gene sizes of the MGEs are represented on the kilobase pair (kb) scale at the bottom. In the ICEs and IME, core genes—including *int*, *int1*, *int2*, *parB*, *rel*, and *rel1*—are highlighted in green, while the remaining essential core genes are shown in black and AMR genes in red. Essential core genes found only in ICEs, such as *topB*, T4SS components, and *mbeC* appear in yellow. All genes were annotated using the NCBI Prokaryotic Genome Annotation Pipeline.

Supplementary Material 5: Supplemental Fig. 2. Phylogenetic tree of *M. haemolytica* isolates, from this study (*n* = 846) and Clawson et at. [8] (*n* = 73), constructed using the refined SNP typing scheme. Isolates classified as genotype 1 (*n* = 118) are marked in green, while genotype 2 isolates (*n* = 801) are marked in purple. The scale bar indicates the number of nucleotide substitutions per site.

## Data Availability

The genomes generated for this study are publicly available through NCBI BioProject accession PRJNA510383: [https://www.ncbi.nlm.nih.gov/bioproject/510383]. The unique SRA accession numbers associated with each sample can be found in Supplemental file 2. Additional data generated and analyzed during this study are included in this published article and supplementary files (1, 2, and 3).

## Abbreviations

AMP: Ampicillin
APHIS: Animal and Plant Health Inspection Service
AMR: Antimicrobial resistance
AST: Antimicrobial susceptibility testing
BRD: Bovine respiratory disease
XNL: Ceftiofur
CLI: Chlortetracycline
DAN: Danofloxacin
ENR: Enrofloxacin
FFN: Florfenicol
GAM: Gamithromycin
GEN: Gentamicin
ICE: Integrative and conjugative element
ICE*Mh1*: Integrative and conjugative element in *M. haemolytica*
ICE*Pmu1*: Integrative and conjugative element in *P. multocida*
IME: Integrative and mobilizable element
MIC: Minimum inhibitory concentration
MGEs: Mobile genetic elements
MDR: Multidrug-resistant
NAHLN: National Animal Health Laboratory Network
NVSL: National Veterinary Services Laboratories
NEO: Neomycin
OXY: Oxytetracycline
PEN: Penicillin
PFGE: Pulsed-field gel electrophoresis
QPM: Quinolone point mutation
SPE: Spectinomycin
SDM: Sulphadimethoxine
TET: Tetracycline
TIA: Tiamulin
TIP: Tildipirosin
TIL: Tilmicosin
SXT: Trimethoprim/Sulfamethoxazole
T4SS: Type IV secretion system
TUL: Tulathromycin
TYLT: Tylosin tartrate
WGS: Whole-genome sequencing
